# Editorial: Advanced catalytic materials and processes in hydrogen technology

**DOI:** 10.3389/fchem.2023.1314796

**Published:** 2023-10-31

**Authors:** Yongxiao Tuo, Wenyao Chen, Nimai Mishra, Bin Wang, Jun Zhang

**Affiliations:** ^1^ State Key Laboratory of Heavy Oil Processing, College of New Energy, China University of Petroleum (East China), Qingdao, Shandong, China; ^2^ State Key Laboratory of Chemical Engineering, East China University of Science and Technology, Shanghai, China; ^3^ Department of Chemistry, SRM University-AP, Amaravathi, Andhra Pradesh, India; ^4^ School of Chemical Engineering and Technology, Xi’an Jiaotong University, Xi’an, Shaanxi, China

**Keywords:** hydrogen, catalysts, catalytic process, reaction kinetics, DFT calculation

## Introduction

Hydrogen is regarded as the ultimate clean energy alternative, offering a sustainable, clean, and efficient source of green energy (Anantharaj et al., 2021). It can be produced not only through the reforming of fossil fuels such as coal, oil, and natural gas, but also from by-product gases in industries like chlor-alkali and metallurgy. Furthermore, green hydrogen can be generated using renewable energy sources like solar, wind, and tidal energy (Li et al., 2020). Hydrogen has multifaceted applications, serving as raw material, reducing agent, and heat source in various industries, while also powering vehicles and ships through fuel cells (Loges et al., 2008; Zhang et al., 2016). This Research Topic mostly emphasizes the evolving landscape of hydrogen technology, encompassing catalytic reaction processes, materials, kinetic analysis, and economic assessments related to hydrogen production, storage and transportation, and applications.

To begin, hydrogen can be created through various methods, including electrocatalytic water splitting and organic liquid fuel steam reforming hydrogen production (Chen et al., 2021; Tuo et al., 2021). Secondly, there is significant progress in hydrogen storage technologies, including compressed gas storage, organic liquid storage, and solid-state storage in the form of hydrides (Lang et al., 2020; Chen et al., 2022; Tarkowski and Uliasz-Misiak, 2022). Finally, the efficient use of hydrogen energy relies heavily on the advancement of hydrogen fuel cell technology in the future (Wang et al.). In conclusion, the development of catalysts and reactions related to the hydrogen economy can catalyze the growth of this field, fostering innovation in catalytic materials and processes and advancing our understanding of catalytic chemistry and surface engineering techniques, thus promoting widespread hydrogen technology development.

## Significant advances in this Research Topic

This Research Topic comprises four research articles, exploring novel catalysts, mathematical modeling, reaction kinetics, and more.

The Oxygen Reduction Reaction (ORR) plays a pivotal role in modern energy storage and conversion systems like fuel cells and metal-air batteries. Unfortunately, ORR faces challenges due to its multi-step, sluggish electron transfer, and low mass transfer efficiency, resulting in slow kinetics. Therefore, there is a pressing need for effective catalysts and a deeper understanding of reaction kinetics to facilitate rapid charge and mass transfer in ORR. The primary structural factors affecting ORR activity is the composition, morphology, particle size, microscopic pore structure of the catalysts. Notably, catalysts with stable structures and larger specific surface areas tend to exhibit enhanced ORR activity. This enhancement is primarily due to the increased exposure of active sites and the enhancement of mass transfer (Zaman et al., 2021). Wang et al. have ingeniously developed a three-dimensional nitrogen-doped carbon material with a layered porous structure, Daikon-NH_3_-900, using biomass and a simple NH_3_ annealing process. This unique structure shortens diffusion paths, minimizes diffusion resistance at the electrode-electrolyte interface, enhances ion transport, and increases active sites, significantly improving ORR performance in both alkaline and acidic conditions. Furthermore, Daikon-NH_3_-900 serves as a high-performing cathode catalyst, achieving a high open circuit voltage of ∼0.98 V, a limiting current density of 1,303 mA·cm^−2^, and a peak power density of 245 W/g. In terms of reaction kinetics, Zhang et al. have developed a mathematical model to explore the relationship between total mass transfer resistance and internal diffusion efficiency factors by introducing carbon nanotubes (CNTs) or graphene oxide (GO) into carbon black (XC-72). Their findings suggest that altering the cathode catalyst layer’s texture can enhance oxygen transport. Smaller carbon materials and Pt nanoparticles can reduce internal oxygen transfer barriers, emphasizing the importance of considering particle size and surface chemical properties of the support when selecting catalyst supports. Additionally, Yao et al. have developed measurement and calculation methods for both in-plane and through-plane oxygen diffusion coefficients using commercial carbon paper GDL (AvCarb EP40) as an example. Furthermore, simulations were conducted to analyze oxygen flux distributions at varying torque and gas flow rate conditions. The findings demonstrate that both gas flow rate and torque exert an influence on both in-plane and through-plane oxygen diffusion coefficients. Specifically, as torque increases and gas flow rate decreases, the in-plane oxygen diffusion coefficient decreases. Their research reveals that gas flow rates and torque influence these diffusion coefficients, providing valuable insights for enhancing fuel cell design and operation.

In the realm of hydrogen storage and transportation, N-ethylcarbazole (NEC) stands out as a promising liquid organic hydrogen carrier because heteroatom-doped heterocyclic compounds are favorable for dehydrogenation reactions. Wang et al. have successfully developed a Ni_4_Mo/AC bimetallic catalyst using an impregnation method for the NEC hydrogenation reaction, significantly improving the selectivity of the final product 12H-NEC. Operating at 150°C and 8 MPa, they achieved a remarkable 100% conversion rate of N-ethylcarbazole in just 4 h, with a hydrogenation rate of 5.77% and a final product selectivity of 98.73%. Ni_4_Mo alloy lowers the dissociation energy of hydrogen, enhancing hydrogenation activity and selectivity. Furthermore, increasing alloying degree and reducing particle size further boosts hydrogenation activity and selectivity.

## Conclusion

In summary, hydrogen energy is pivotal in promoting economic and industrial development, with its future prospects heavily reliant on advancing hydrogen technology. This Research Topic focuses on showcasing the latest advancements in catalytic materials and processes in hydrogen technology, which are expected to play a significant role in advancing hydrogen production, storage and transportation, and application technologies. We extend our heartfelt gratitude to the editorial board, authors, and reviewers for their invaluable support of this Research Topic.
